# Methylprednisolone Decreases Mitochondria-Mediated Apoptosis and Autophagy Dysfunction in Hepatocytes of Experimental Autoimmune Hepatitis Model *via* the Akt/mTOR Signaling

**DOI:** 10.3389/fphar.2019.01189

**Published:** 2019-10-18

**Authors:** Xiaoli Fan, Ruoting Men, Haoran Wang, Mengyi Shen, Tingting Wang, Tinghong Ye, Xuefeng Luo, Li Yang

**Affiliations:** ^1^Department of Gastroenterology and Hepatology, Sichuan University-Oxford University Huaxi Gastrointestinal Cancer Centre,West China Hospital, Sichuan University, Chengdu, China; ^2^Laboratory of Liver Surgery, State Key Laboratory of Biotherapy/Collaborative Innovation Center for Biotherapy, West China Hospital, Sichuan University, Chengdu, China

**Keywords:** autoimmune hepatitis, methylprednisolone, LO2 cell, apoptosis, autophagy, Akt/mTOR signaling pathway

## Abstract

Autoimmune hepatitis (AIH) is characterized by massive immune cell-mediated hepatocyte destruction. Glucocorticoids, particularly methylprednisolone (MP), are the most effective treatment for AIH; however, the mechanism underlying the effects of glucocorticoid treatment has not been fully elucidated. The present study explored the effects of MP on damaged hepatocytes in mice with concanavalin A (ConA)−induced experimental autoimmune hepatitis (EAH). C57BL/6 mice were divided into three groups: a normal control group (injected with normal saline), a ConA (20 mg/kg) group, and a ConA + MP (3.12 mg/kg) group. The serum levels of liver enzymes, cytokines, activated T cells, and apoptosis− and autophagy−associated marker proteins were determined 12 h after ConA injection. Human hepatocyte cell line LO2 was used to verify the effects of ConA and MP *in vitro*. MP treatment significantly decreased inflammatory reactions in the serum and liver tissues and activated the Akt/mTOR signaling pathway to inhibit apoptosis and autophagy in hepatocytes *in vivo*. Transmission electron microscopy (TEM) revealed fewer autophagosomes in the MP-treated group than in the ConA-treated group. MP treatment obviously suppressed apoptosis and mitochondrial membrane potential (ΔΨm) loss in hepatocytes *in vitro*. Furthermore, ConA treatment increased the levels of LC3-II, p62/SQSTM1, and Beclin-1, while bafilomycin A1 did not augment the levels of LC3-II. MP treatment decreased the levels of LC3-II, p62/SQSTM1, and Beclin-1 and upregulated the levels of phosphorylated (p)-Akt and p-mTOR. In conclusion, MP ameliorated mitochondria-mediated apoptosis and autophagy dysfunction in ConA-induced hepatocyte injury *in vivo* and *in vitro via* the Akt/mTOR signaling pathway.

## Introduction

Autoimmune hepatitis (AIH) is a self-perpetuating inflammatory liver disease, and the misdiagnosis or delayed treatment of AIH can lead to liver cirrhosis, liver cancer, transplantation, and rapid death (European Association for the Study of the Liver, 2015; Manns et al., 2015; Montano-Loza and Czaja, 2015).

Methylprednisolone (MP) is a physiological inhibitor of inflammatory responses that is widely used as an anti-inflammatory and immunosuppressive agent in the treatment of numerous autoimmune and allergic diseases, mainly through suppressing CD4+ T cell activation indirectly by modulating dendritic cell function and directly by regulating T cell receptor signaling (Cain and Cidlowski, 2017). Glucocorticoids have been reported to prevent the progression of various liver diseases by the following mechanisms: (i) protection of normal human liver cells from apoptosis induced by tumor necrosis factor (Zhao et al., 2015), (ii) suppression of calpain μ activation and talin degradation in ischemia-induced liver injury in rats (Wang et al., 2008), (iii) increased ability of hepatocytes to accumulate cAMP due to protein synthesis-dependent processes (Thoresen et al., 1989). Longhi *et al*. found that corticosteroids used to treat AIH can reconstitute the regulatory T cell population, which, in turn, suppresses the proliferation of CD8+ lymphocytes and induces production of the anti-inflammatory cytokine IL-4 (Longhi et al., 2005). Furthermore, Moser *et al*. revealed that glucocorticoids affect the capacity of dendritic cells to sensitize naive T cells *in vivo* (Moser et al., 1995). However, the mechanisms by which glucocorticoids alleviate AIH remain to be elucidated.

Apoptosis and autophagy, which are overactivated following ConA treatment, contribute to liver injury (Yin et al., 2008; Czaja, 2014; Li et al., 2016c; Feng et al., 2018b). Apoptosis, the predominant mechanism of liver cell death in interface hepatitis (Kerr et al., 1979), is a double-edged sword, as it can both inhibit inflammatory responses and lead to tissue damage when overactivated. Autophagy is an evolutionarily conserved process that plays an important role in responses to different types of cellular stress (Yu et al., 2018). We hypothesized that MP may ameliorate the pathological changes in liver injury by regulating apoptosis and autophagy in hepatocytes and subsequently relieve AIH progression. The present study was designed to evaluate whether MP affects the levels of apoptosis and autophagy *in vivo* and *in vitro* and the possible mechanism underlying this effect.

## Materials and Methods

### Drugs and Reagents

ConA was purchased from Sigma–Aldrich (St. Louis, MO, USA). MP (HY-B0260, analytical standard >99.0%) and bafilomycin A1 (HY-100558, analytical standard >99.0%) were obtained from MedChemExpress (NJ, USA). Antibodies p62/SQSTM1 (cat# 5114), Beclin-1 (cat# 3495), p-mTOR (Ser 2448, cat# 5536), mTOR (cat# 2983), p-Akt (Ser 473, cat# 4060), Akt (cat# 4691), Bax (cat# 2772), Bcl-2 (cat# 3498), and cleaved caspase-3 (cat# 9664) were purchased from Cell Signaling Technology (Danvers, MA, USA). LC3B (sc-376404) was purchased from Santa Cruz Biotechnology (Europe), and β-actin was purchased from ZSGB-BIO (Beijing, China). Monodansylcadaverine (MDC, G0170) was purchased from Solarbio (Beijing, China). The tetraethylbenzimidazolylcarbocyanine iodide (JC-1) staining kit was purchased from Beyotime (Shanghai, China). Other reagents were of high analytical grade and were commercially available unless otherwise specified.

### Animals

Female C57BL/6 mice (aged 8–10 weeks; 19–22 g) were obtained from the Laboratory Animal Center of Chongqing Medical University (Chongqing, China). The mice were housed in a specific pathogen-free (SPF) facility at a constant room temperature and humidity and had unlimited access to standard laboratory chow and water one week before the experiments. All animal experiments were approved by the Institutional Animal Care and Treatment Committee of Sichuan University in China.

### Experimental Design and Treatment Schedule

**Figure 1:**
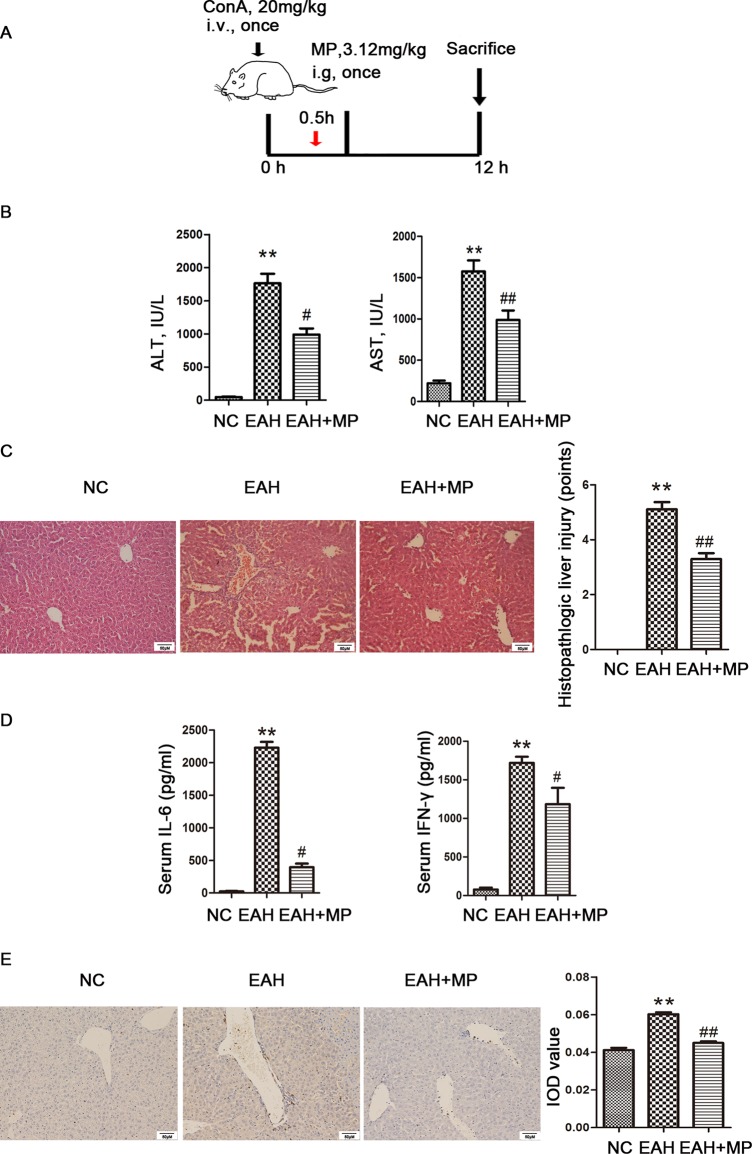
Effects of MP on ConA−induced EAH. **(A)** Animal study protocol. **(B)** Serum ALT and AST. **(C)** Pathological liver specimens stained with H&E (×200): The control group showed normal hepatic architecture, while ConA injection induced marked pathological damage, including dramatic inflammatory cell infiltration in the portal area, massive cloudy swelling, blood vessel congestion and dilatation, and disordered hepatic sinusoid structures. MP-treated animals showed improvements of these lesions. **(D)** Serum IL-6 and IFN-γ. **(E)** Immunohistochemistry was used to show CD4+ T cell infiltration (original magnification, ×200). ConA injection in the EAH group induced a significant increase in the number of infiltrating CD4+ T cells compared to that in the control group. The percentage of CD4+ T cells was significantly decreased after MP treatment and almost returned to normal levels compared to that in the EAH group. Data are presented as the mean ± SD (n = 8). **P < 0.01 vs. control group; ^#^P < 0.05, ^##^P < 0.01 vs. EAH group. ConA, concanavalin A; MP, methylprednisolone; NC, normal control; EAH, experimental autoimmune hepatitis; ALT, alanine aminotransferase; AST, aspartate aminotransferase; IL-6, interleukin-6; IFN-γ, interferon-γ; IOD, integrated optical density.

An experimental autoimmune hepatitis (EAH) mouse model was established 12 h after the injection of ConA at a dose of 20 mg/kg, which was chosen based on our previous studies (Wang et al., 2018a; Ye et al., 2018). The animals were randomly divided into three groups (n = 8 per group): (1) a normal control group (NC), (2) an EAH group (EAH), and (3) an EAH group with MP (3.12 mg/kg). The distribution of mice in all the groups was random. Mice in the MP and NC groups received MP or saline by intragastric administration once 0.5 h after the ConA injection. Finally, all of the mice were sacrificed 12 h after the ConA injection (**Figure 1A**).

### Liver Function and Cytokine Assay

Retro-orbital blood samples were collected by removing the eyeball. Plasma was separated by centrifugation at 2,000 rpm for 10 min, and alanine transaminase (ALT) and aspartate transaminase (AST) measurements were performed using an automatic dry biochemical analyzer (Hitachi Auto Analyzer7170, Japan). The levels of IL-6 and IFN-γ in the murine plasma were analyzed by ELISA kits (MultiSciences, Hangzhou, China) according to the manufacturer’s instructions.

### Histopathological Analysis

Liver tissues were isolated, and the samples were fixed in 4% buffered paraformaldehyde for 48 h and then embedded in paraffin. The sections (4–5 μm) were mounted on slides, deparaffinized in xylene, rehydrated in decreasing concentrations of ethanol, and subjected to hematoxylin and eosin (H&E) staining.

### Immunohistochemistry (IHC) Analysis

Paraffin-embedded tissue samples were dewaxed, rehydrated, and incubated with a primary antibody overnight at 4°C. The samples were incubated with a secondary antibody (Bioss, Beijing, China) according to the manufacturer’s instructions. A brown color in the cell membrane indicated positive staining. The integrated optical densities (IODs) of the different indicators were calculated using Image-Pro Plus software 6.0 (Media Cybernetics, Silver Spring, MD, USA).

### TUNEL Assay

A TUNEL assay was performed according to the protocol provided in the TUNEL kit. Paraffin-embedded liver sections sliced at a 5-μm thickness were dewaxed in xylene for 10 min twice and dehydrated in ethanol. Then, the sections were incubated with 20 μg/ml DNase-free proteinase K at room temperature for 15 min to digest the sections. After washing 4 times, a TUNEL reaction mixture was added to the sections, which were then incubated at 37°C in a humidified atmosphere for 1 h and observed by light microscopy.

### Transmission Electron Microscopy (TEM)

Fragments from the liver parenchyma of each mouse were fixed in 2.5% glutaraldehyde, fixed in 1% osmium acid, dehydrated in an alcohol gradient, embedded in epoxy resin, and sliced by a microtome. The sections were stained, and images were taken by TEM (HT7700, Hitachi, Japan). For quantification of autophagosomes in hepatocytes of each group, 10 lower-magnification photomicrographs from the hepatocytes of hepatic tissues (×2,500, each image containing at least one hepatocytes nucleus) were applied (Watanabe et al., 2009; Eid et al., 2013).

### Cell Culture and Treatments

Human foetal hepatocyte cell line LO2 was cultured in DMEM supplemented with 10% fetal bovine serum (FBS) and a 1% penicillin-streptomycin solution at 37°C in a humidified 5% CO_2_ incubator. To induce excessive inflammation and develop an *in vitro* model of hepatocyte injury, LO2 cells were exposed to medium supplemented with ConA for 12 h. To test the effects of MP, cells were incubated with 10 μM MP 2 h before ConA treatment and then incubated with bafilomycin A1 (10 nM) 3 h before harvesting. ConA and MP were dissolved in saline and distilled water, respectively, and bafilomycin A1 was dissolved in DMSO according to the manufacturer’s instructions.

### Cell Proliferation Assay

Cells (2-5×10^3^/100 μl/well) were seeded in 96-well plates and treated with different concentrations of MP for 12 h. Next, 20 μl of MTT solution (5 mg/ml) was added to each well, and the cells were continuously incubated for 4 h. The purple formazan crystal was dissolved in 150 μl of DMSO, and the absorbance at 570 nm was subsequently recorded using a Spectra MAX M5 microplate spectrophotometer (Molecular Devices, CA, USA).

### Morphological Analysis by Hoechst 33258 Staining

To conduct staining experiments, LO2 cells at 50% confluence were plated on 18-mm coverslips in a six-well plate and incubated for 12 h. After treatment with the different reagents for 12 h, the cells were washed with ice-cold phosphate-buffered saline (PBS) twice and fixed in ice-cold methanol for approximately 15 min. The cells were stained with a Hoechst 33258 solution (KeyGen Biotech) and then photographed by a fluorescence microscope to observe the nuclear morphology of the apoptotic bodies.

### Apoptosis Analysis by Flow Cytometry (FCM)

Further observation of apoptosis was carried out using an Annexin V-FITC/PI apoptosis detection kit (KeyGEN BioTECH). After treatment with the different reagents, the cells were collected, washed twice with ice-cold PBS, and then stained with Annexin V-FITC and PI according to the manufacturer’s guidelines. The distributions of viable (FITC-/PI-), early-apoptotic (FITC+/PI-), late-apoptotic (FITC+/PI+), and necrotic (FITC-/PI+) cells were analyzed *via* FCM. Both early- and late-apoptotic cells were defined as apoptotic cells in this study. Data were analyzed using FlowJo software according to the manufacturer’s instructions.

### Examination of Mitochondrial Membrane Potential (Δψm) and Reactive Oxygen Species (ROS)

To detect changes in the ΔΨm and ROS level, LO2 cells treated with the different reagents were incubated with 10 μM rhodamine 123 (Rh123) and 2’,7’-dichlorodihydrofluorescein diacetate (DCFH-DA) diluted in PBS at 37°C in the dark for 30 min. Finally, the stained cells were washed with cold PBS and analyzed *via* FCM. The data are shown as the mean values from independent experiments that were conducted in triplicate.

ΔΨm was further verified by tetraethyl benzimidazolyl carbocyanine iodide (JC-1) staining (Pourahmad et al., 2003). LO2 cells were seeded at a density of 5 × 10^4^ cells/well on coverslips and incubated in 24-well plates. Before incubation with ConA for 12 h, the cells were pretreated with 10 μM MP for 2 h. After drug treatment, the cells were washed with PBS and incubated in medium containing 5 g/ml JC-1 stain for 20 min in the dark at 37°C. The cells were then directly observed under a fluorescence microscope at an excitation wavelength of 514–529 nm and an emission wavelength of 585–590 nm.

### Monodansylcadaverine (MDC) Staining

Autophagic vacuoles formed in the cells were detected by MDC staining. LO2 cells were seeded at a density of 5 × 10^4^ cells/well on cover slips in 24-well plates and incubated overnight to allow adherence. Then, the cells were treated with saline or ConA (10 μg/ml) alone or in combination with MP for 12 h. The cells were then washed three times with PBS and incubated with MDC (50 μM) for 30 min in the dark at 37°C. Next, excess MDC was washed away, and the cells on the cover slips were washed with PBS and fixed with 4% paraformaldehyde for 15 min. Autophagic vacuoles formed in the cells were analyzed by fluorescence microscopy at an excitation wavelength of 460–500 nm and an emission wavelength of 512–542 nm.

### Western Blot Analysis

Liver specimens and LO2 cells were lysed using RIPA lysis buffer in the presence of protease and phosphatase inhibitors for 30 min on ice, followed by centrifugation at 13300 rpm at 4°C for 15 min to clear the lysates; the lysate supernatant was then harvested. Protein concentrations were determined with the Bradford Protein Assay Kit, and known amounts of BSA were used to standardize and equalize protein concentrations before loading. Equivalent amounts of total protein (usually 30–40 μg) were separated on SDS-PAGE gels and then transferred to polyvinylidene difluoride (PVDF) membranes (Amersham Bioscience, Piscataway, NJ). After blocking, the membranes were incubated overnight at 4°C with primary antibodies including cleaved caspase-3, Bax, Bcl-2, LC3B, Beclin-1, p62, Beclin-1, Akt, p-Akt, mTOR, p-mTOR, and β-actin. After washing with TBST, the membranes were incubated with an HRP-conjugated secondary antibody (ZSBIO, Beijing, China) at 37°C for 1 h. Finally, the membranes were washed twice and detected using an enhanced chemiluminescence system. The IODs of the different indicators were calculated using Image-Pro Plus software.

### Statistical Analysis

Descriptive and analytical statistical analyses were performed using the SPSS (version 22.0) software package. Statistical significance between groups was determined by a two-tailed Student’s *t*-test. The data are presented as the mean ± SD, and P < 0.05 indicates statistical significance.

## Results

### MP Treatment Attenuates Liver Injury Induced by ConA in Mice

An important characteristic of AIH is an obviously elevated serum level of transaminase, which indicated the extent of liver injury. A ConA-induced injury model was established as previously described with or without treatment with MP (3.12 mg/kg), as shown in **Figure 1A**. We determined the levels of liver enzymes in mouse serum 12 h after ConA injection. As shown in **Figure 1B**, the liver enzyme levels in the EAH group were significantly higher than those in the NC group, indicating the successful simulation of human AIH. In addition, the ALT and AST levels were reduced by MP treatment, demonstrating the protective effect of MP in the EAH liver. As the progression of liver injury is associated with proinflammatory cytokines, the serum levels of IL-6 and IFN-γ were examined and determined by ELISA (**Figure 1D**). As expected, the levels of these cytokines in the EAH group were significantly higher than those in the NC group and were reduced by MP treatment.

We then examined the effect of MP on EAH liver damage using H&E staining. As shown in **Figure 1C**, mice in the EAH group suffered severe liver damage, as indicated by massive inflammatory cell infiltration in areas around the central vein. In contrast, those in the EAH+MP group exhibited minor liver injury, indicating that treatment with MP significantly reduced liver injury.

As the T cell-mediated immune response plays a vital role in AIH, the T cell infiltration in the livers in the different groups were analyzed by IHC. As indicated by CD4 staining, portal areas showed T cell infiltration upon ConA treatment, while MP treatment decreased the presence of T cells in the liver tissues (**Figure 1E**).

The above data verified that MP treatment alleviates inflammation and hepatocyte injury and changes the T cell-mediated immune response in the liver.

### MP Treatment Attenuates Hepatocyte Apoptosis and Autophagy in ConA-Induced Liver Injury

**Figure 2:**
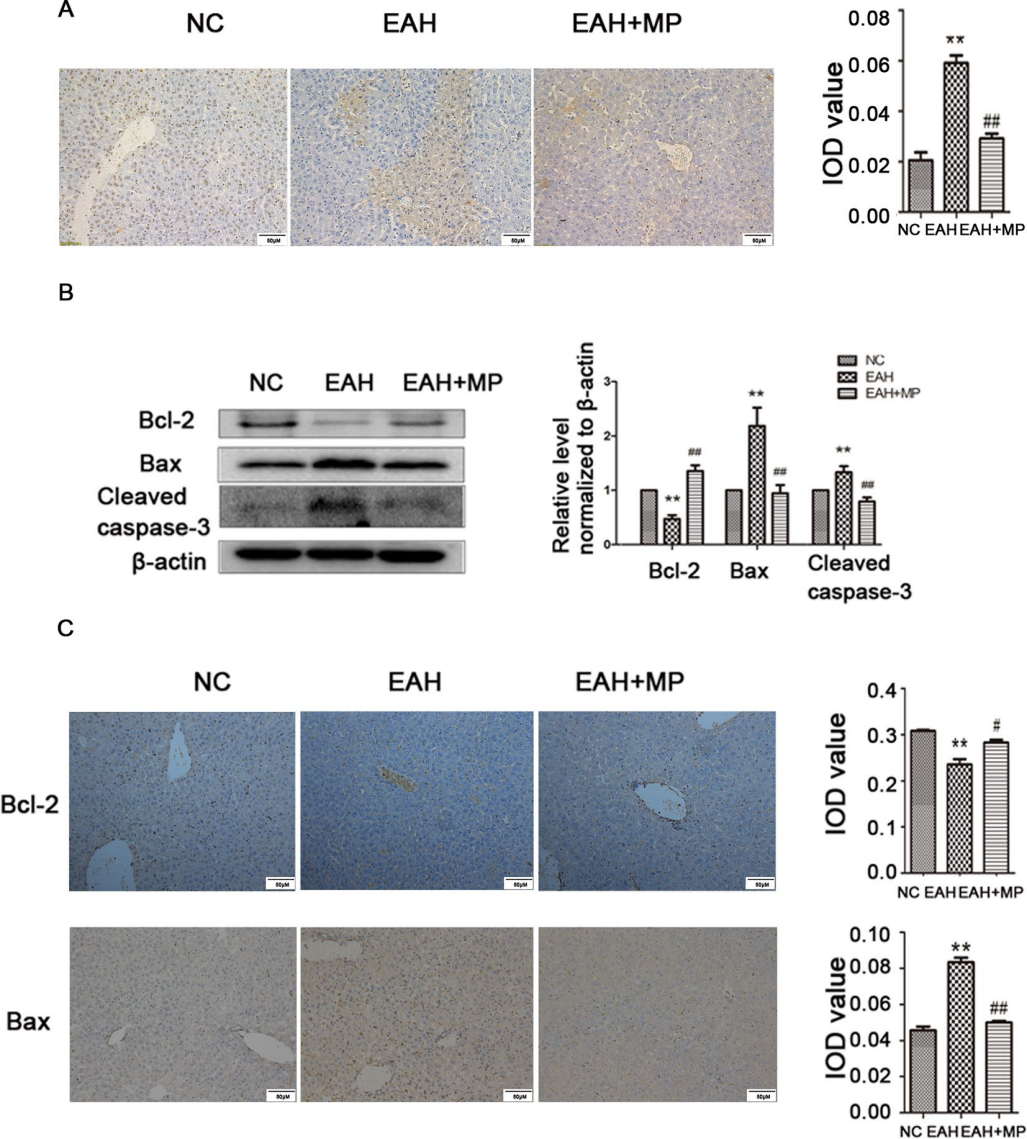
MP pretreatment attenuates hepatocyte apoptosis and autophagy in ConA−induced EAH, partially through the Akt/mTOR signaling pathway. **(A)** TUNEL staining indicated apoptotic hepatocytes in the three groups after ConA injection (original magnification, ×400). **(B)** Protein expression of Bcl−2, Bax, and cleaved caspase−3 in liver tissues. **(C)** Immunohistochemistry was used to detect Bcl−2 and Bax levels in liver tissues (original magnification, ×200). ConA, concanavalin A; MP, methylprednisolone; NC, normal control; EAH, experimental autoimmune hepatitis; IOD, integrated optical density. **P < 0.01 vs. NC group; ^#^P < 0.05 vs. ConA group (20 mg/kg); ^##^P < 0.01 vs. ConA group (20 mg/kg).

Hepatocyte apoptosis was strongly induced in the EAH group compared to the control, while MP reduced this effect as shown by a reduction in the TUNEL positive staining (**Figure 2A**). This finding was further supported by analyzing the indicators of cellular apoptosis. B-cell lymphoma-2 (Bcl-2) is an antiapoptotic protein, while Bcl-2-associated X protein (Bax) and caspase-3 are markers of apoptosis. In the EAH group, the expression of cleaved caspase-3 and Bax was increased, while the production of Bcl-2, which inhibits apoptosis, was dramatically reduced compared to that in the NC group. Conversely, MP treatment downregulated the proapoptotic markers Bax and cleaved caspase-3 and upregulated the expression of Bcl-2 (**Figure 2B**). The IHC staining results were consistent with those of western blot (**Figure 2C**). The results of the TUNEL assay and apoptosis-related protein analysis implied that ConA-induced apoptosis in hepatocytes and that MP treatment alleviated apoptosis.

**Figure 3:**
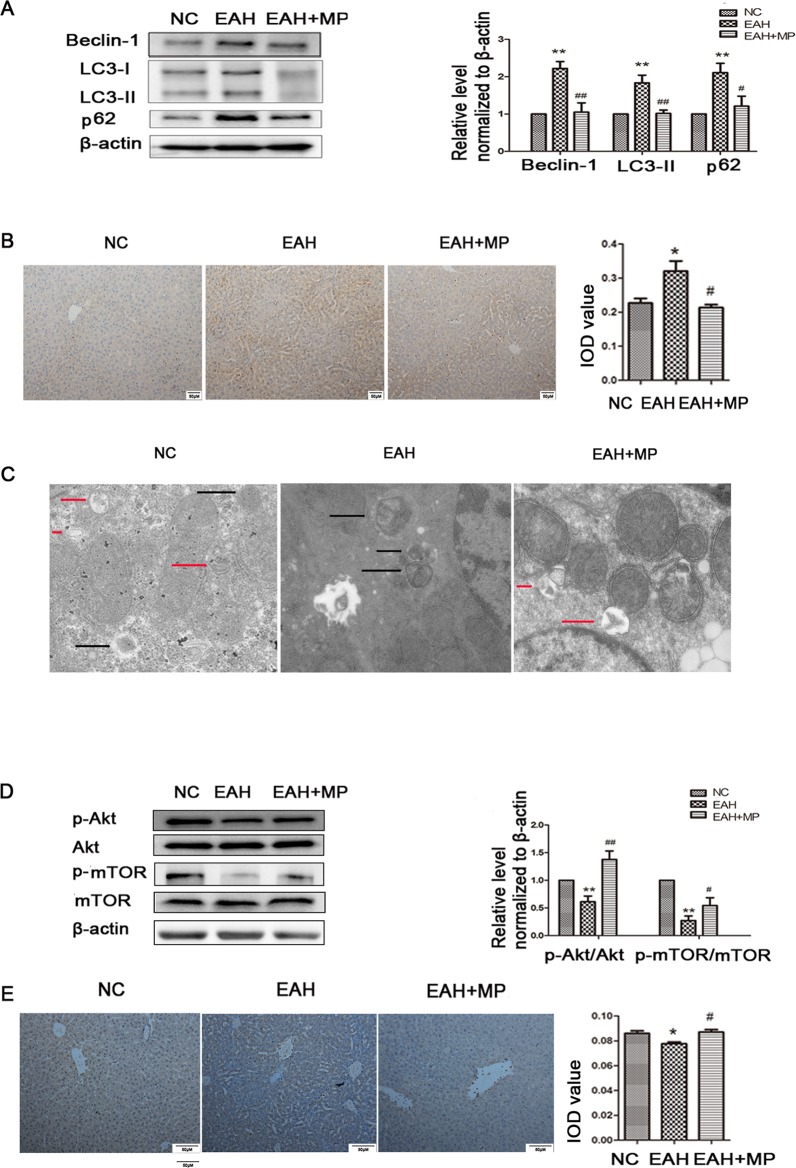
**(A)** Protein expression of beclin−1, LC3 and p62. **(B)** Immunohistochemistry was used to detect LC3 levels in liver tissues (original magnification, ×200). **(C)** Transmission electron microscopy examination of autophagosomes and autolysosomes the hepatocytes of hepatic tissues. Black arrows denote autophagosomes and red arrows denote autolysosomes (original magnification, ×7k). **(D)** Protein expression of Akt, p-Akt and p-mTOR, mTOR. **(E)** Immunohistochemistry was used to detect p−Akt levels in liver tissues (original magnification, ×200). In western blot analyses, β−actin was used as the internal control; the relative protein expression level in the treated group was calculated after setting the control group expression (0 μg/ml) value to 1.00. Data are presented as the mean ± SD. *P < 0.05 vs. NC group; **P < 0.01 vs. NC group; ^#^P < 0.05 vs. EAH group; ^##^P < 0.01 vs. EAH group. ConA, concanavalin A; MP, methylprednisolone; NC, normal control; EAH, experimental autoimmune hepatitis; IOD, integrated optical density.

LC3-B, p62, and Beclin-1 are markers of autophagy. Western blot analyses indicated that ConA injection significantly upregulated the LC3-II, Beclin-1, and p62 expression levels compared with those in the NC group. Treatment with MP reversed this effect (**Figure 3A**), which was further confirmed by IHC staining (**Figure 3B**). As shown in **Figure 3C**, TEM images showed more autophagosomes in the EAH group than in the NC group; conversely, MP treatment decreased the number of autophagosomes (**Supplementary Figure 1**).

These results provide strong evidence that MP treatment attenuates hepatocyte apoptosis and autophagosomes and reduces pathological liver damage following ConA administration in mice.

### Pretreatment With MP Ameliorates Liver Injury in EAH At Least in Part Through the Akt/mTOR Signaling Pathway

As mentioned above, MP treatment protected against liver injury by inhibiting hepatocyte apoptosis and autophagosomes. However, the mechanism underlying this effect remains unclear. To determine whether the protective effect of MP on EAH injury is mediated by the Akt/mTOR signaling pathway, the protein levels of Akt, p-Akt, mTOR, and p-mTOR in liver tissues were detected. ConA injection downregulated the p-Akt/Akt and p-mTOR/mTOR levels in the liver tissues of the EAH group. MP treatment, however, partly suppressed this effect, which was significant (**Figure 3D**). Similar results were observed for p-Akt in the IHC analysis (**Figure 3E**). In summary, MP treatment may activate the Akt/mTOR signaling pathway in ConA-induced liver injury.

### ConA Treatment Aggravates Apoptosis and Autophagy Dysfunction in LO2 Cells

**Figure 4:**
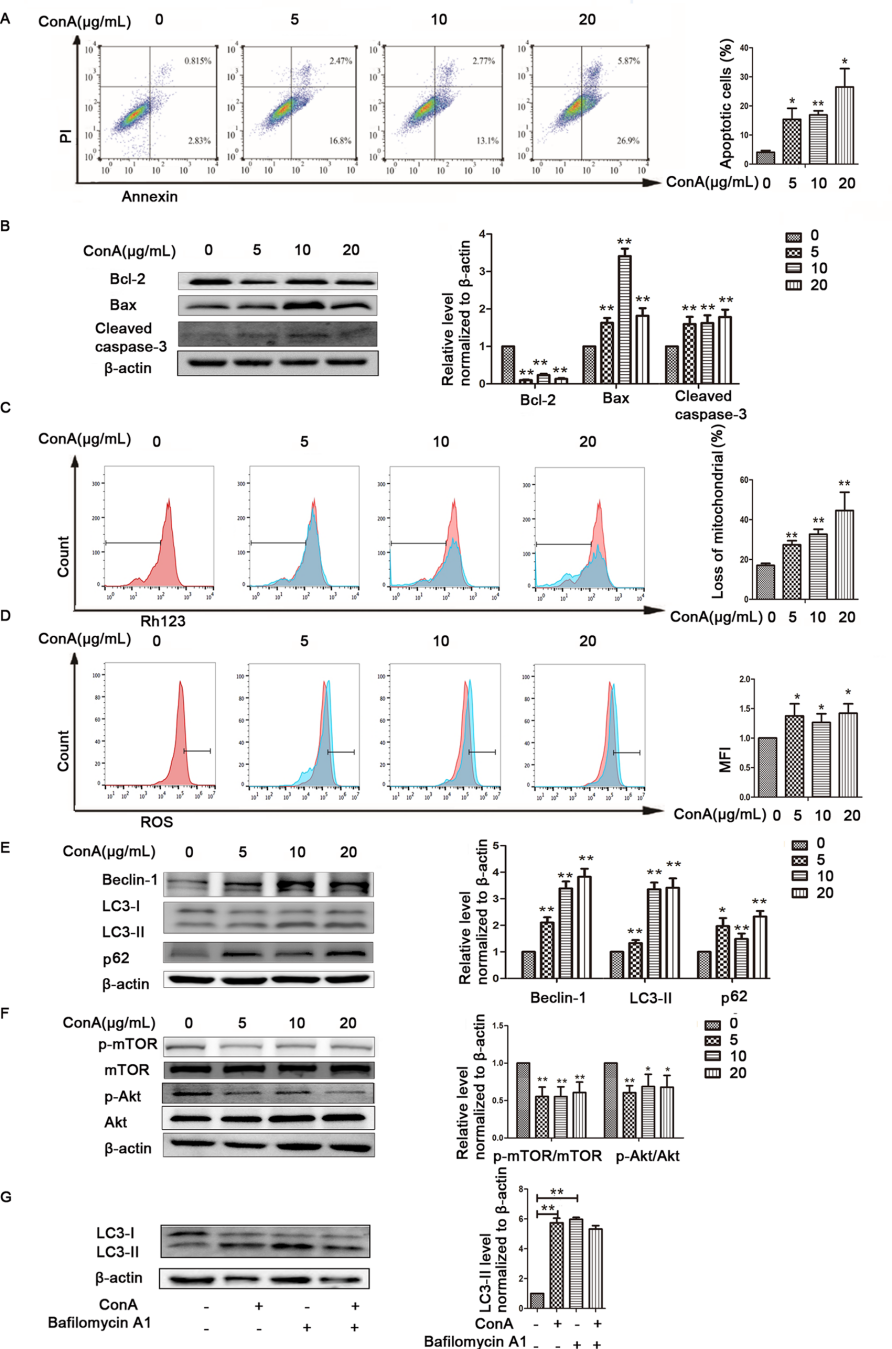
ConA treatment aggravated mitochondria-mediated apoptosis and autophagy dysfunction in LO2 cells. **(A)** The Annexin V and PI dual-labeling technique was used to analyze the effect of ConA treatment on LO2 cell apoptosis. **(B)** The protein expression of Bcl−2, Bax, and cleaved caspase−3 was measured by western blot. **(C)** LO2 cells were treated with various concentrations of ConA for 12 h and then incubated with 10 μM Rh123. The red branches represent the control. **(D)** LO2 cells treated with various concentrations of ConA for 12 h were incubated with 10 mM DCFH-DA for 30 min, and ROS levels were then analyzed by flow cytometry. The red branches represent the control; the relative MFI level in the treated group was calculated after setting the control group expression (0 μg/ml) value to 1.00. **(E)** The protein expression of beclin−1, LC3 and p62 was measured by western blot. **(F)** The protein expression of Akt, p-Akt and p-mTOR, mTOR was measured by western blot. In western blot analyses, β−actin was used as the internal control; the relative protein expression level in the treated group was calculated after setting the control group expression (0 μg/ml) value to 1.00. **(G)** Autophagic flux was calculated by dividing the levels of LC3-II in the presence of bafilomycin A1 by that without bafilomycin A1.*P < 0.05 vs. control group; **P < 0.01 vs. control group. ConA, concanavalin A; MFI, mean fluorescence intensity; DCFH-DA, dichlorodihydrofluorescein diacetate.

The animal experimental results suggested that the mechanism underlying MP treatment needed to be further explored *in vitro*. LO2 cells treated with ConA for 12 h were stained with Annexin V-FITC/PI, and the levels of apoptosis were determined. As shown in **Figure 4A**, the mean apoptosis rates (from three independent experiments) were 4.0% (0 μg/ml ConA group), 15.4% (5 μg/ml ConA group), 16.9% (10 μg/ml ConA group) and 26.5% (20 μg/ml ConA group). LO2 cell apoptosis significantly increased as the dose of ConA increased. Furthermore, the apoptosis-related proteins Bax, Bcl-2, and cleaved caspase-3 in LO2 cells were examined *via* western blot, as shown in **Figure 4B**. The expression of Bcl-2, an antiapoptotic marker, was dramatically reduced, while that of Bax and cleaved caspase-3 was increased.

To investigate whether ConA disrupts ΔΨm, the mitochondria-specific and voltage-dependent dye Rh123 was used to monitor alterations in ΔΨm in LO2 cells. As displayed in **Figure 4C**, ΔΨm in the three ConA-treated groups was vitally different from that in the NC group. Previous studies demonstrated that ROS levels are substantially affected by the inhibition of mitochondrial function (Li et al., 2016b; Yang et al., 2018). We further evaluated the ROS levels in LO2 cells after ConA treatment by DCFH-DA staining. As shown in **Figure 4D**, the ROS levels in LO2 cells treated with ConA were observably increased compared to those in untreated cells.

The effects of ConA on the expression of LC3-II, p62, and Beclin-1 in LO2 cells were detected by western blot. After treatment with 0-20 μg/ml ConA for 12 h, the protein levels of the autophagy markers, including LC3-II, Beclin-1, and p62, were upregulated (**Figure 4E**).

Additionally, the expression levels of p-Akt/Akt and p-mTOR/mTOR were significantly decreased in LO2 cells treated with ConA compared to those in untreated cells (**Figure 4F**). Hence, 10 μg/ml ConA was used for subsequent *in vitro* experiments.

Bafilomycin-A, a specific inhibitor of vacuolar-type H+ ATPase, which inhibits late stages of autophagy by preventing autophagosome-lysosome fusion was used to monitor the effect of ConA on autophagy flux (**Figure 4G**) (**Supplementary Figure 2**). The LC3-II levels were not further augmented in the presence of bafilomycin-A1 treatment indicating that the upregulation of LC3-II was not caused by activation of autophagy pathway, but due to possible impairment of autophagy flux.

### The Protective Effects of MP Treatment on Mitochondria-Mediated Apoptosis and Autophagy in LO2 Cells

**Figure 5:**
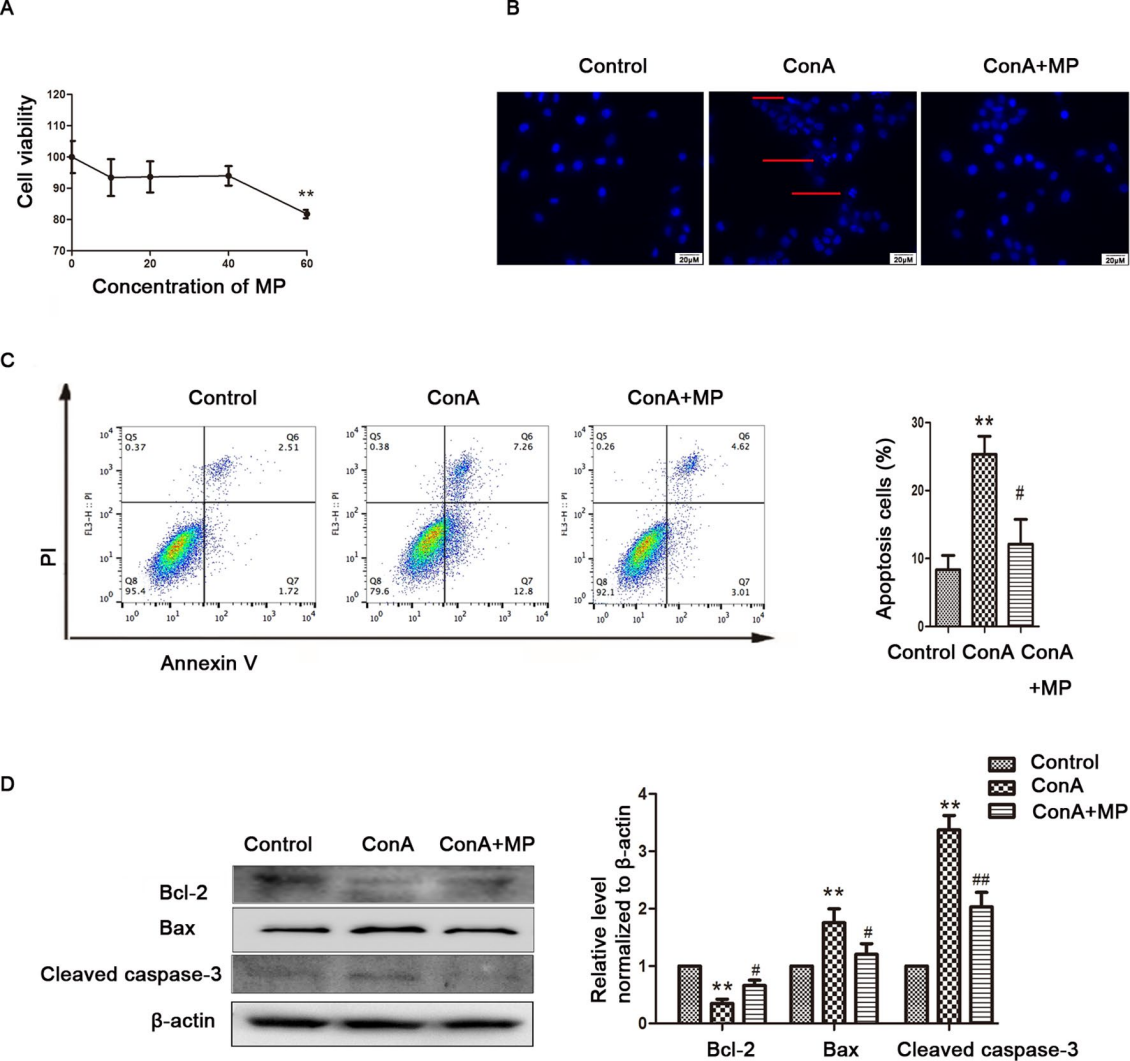
The protective effects of MP treatment on mitochondria-mediated apoptosis in LO2 cells. **(A)** LO2 cells were incubated with the indicated concentrations (10-60 μM) of MP for 12 h. Cell viability was determined by the MTT assay. **(B)** The appearance of apoptotic bodies in the fluorescence microscopy Hoechst 33258 staining assay of LO2 cells. Red arrows denote apoptotic bodies (original magnification, ×400). **(C)** The Annexin V and PI dual-labeling technique was used to analyze the effect of MP treatment on LO2 cell apoptosis. **(D)** The protein expression of Bcl−2, Bax, and cleaved caspase−3 was measured by western blot. β−actin was used as the internal control. The relative protein expression level in the treated group was calculated after setting the control group expression value to 1.00. *P < 0.05 vs. control group; **P < 0.01 vs. control group; ^#^P < 0.05 vs. ConA group; ^##^P < 0.01 vs. ConA group. ConA, concanavalin A; MP, methylprednisolone. **P<0.01 vs. normal control group; ^#^P < 0.05, ^##^P < 0.01 vs. EAH group

**Figure 6:**
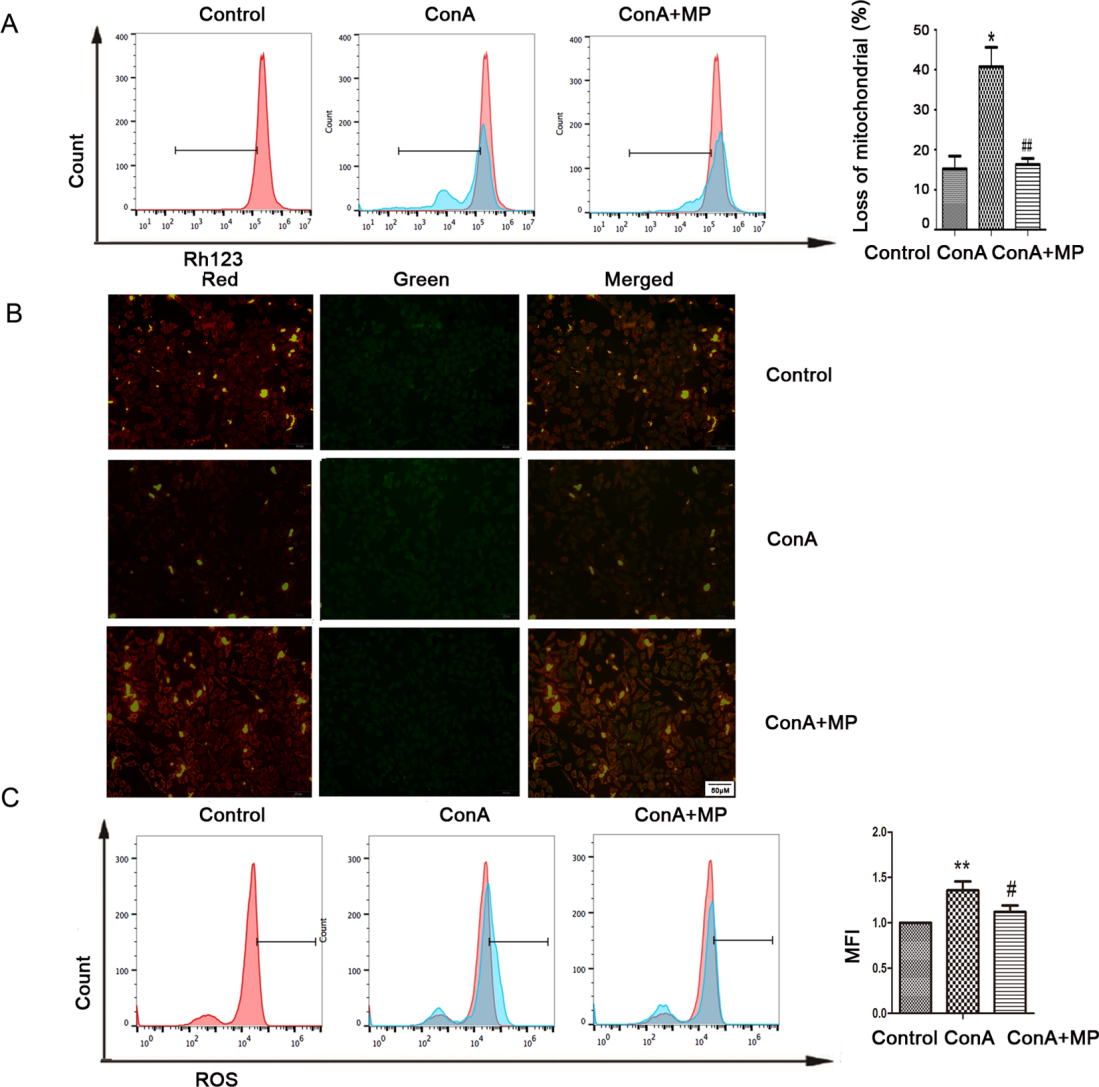
Variation in the Ψm and ROS level. **(A)** LO2 cells were treated with ConA with or without MP for 12 h. The red branches represent the control. Data are shown as the mean ± SD, *P < 0.05 vs. control group; # P < 0.05 vs. ConA group; **(B)** The Δψm (red/green) alteration in LO2 cells was determined by fluorescence microscopy analysis after staining with JC-1. **(C)** LO2 cells treated with ConA with or without MP for 12 h were incubated with 10 mM DCFH-DA for 30 min, and the ROS level was then analyzed by flow cytometry. The control groups are presented as lines filled with red color; the relative MFI level in the treated group was calculated after setting the control group expression (0 μg/ml) value to 1.00. Data are shown as the mean ± SD, *P < 0.05 vs. control group; **P < 0.01 vs. control group; ^#^P < 0.05 vs. ConA group; ^##^P < 0.01 vs. ConA group. ConA, concanavalin A; MP, methylprednisolone; Ψm, mitochondrial membrane potential; ROS, reactive oxygen species; MFI, mean fluorescence intensity.

The *in vitro* results were the same as those observed in liver tissues described above. First, hepatocyte viability following treatment with MP was determined by the MTT assay. MP caused a dose-dependent decrease in LO2 cell viability (**Figure 5A**). Thus, 10 μM MP was chosen for subsequent *in vitro* experiments. The variation in apoptosis was evaluated by Hoechst 33258 staining. After LO2 cells were treated with ConA with or without MP for 12 h, several apoptotic bodies were observed in the ConA group, whereas MP treatment decreased apoptosis (**Figure 5B**). The FCM and western blot results indicated that ConA treatment significantly induced mitochondria-mediated apoptosis; however, pretreatment with MP significantly reduced apoptosis (**Figures 5C, D**). The results of ΔΨm transformation and the ROS level also verified that MP pretreatment may improve mitochondria-mediated apoptosis in LO2 cells (**Figures 6A–C**).

**Figure 7:**
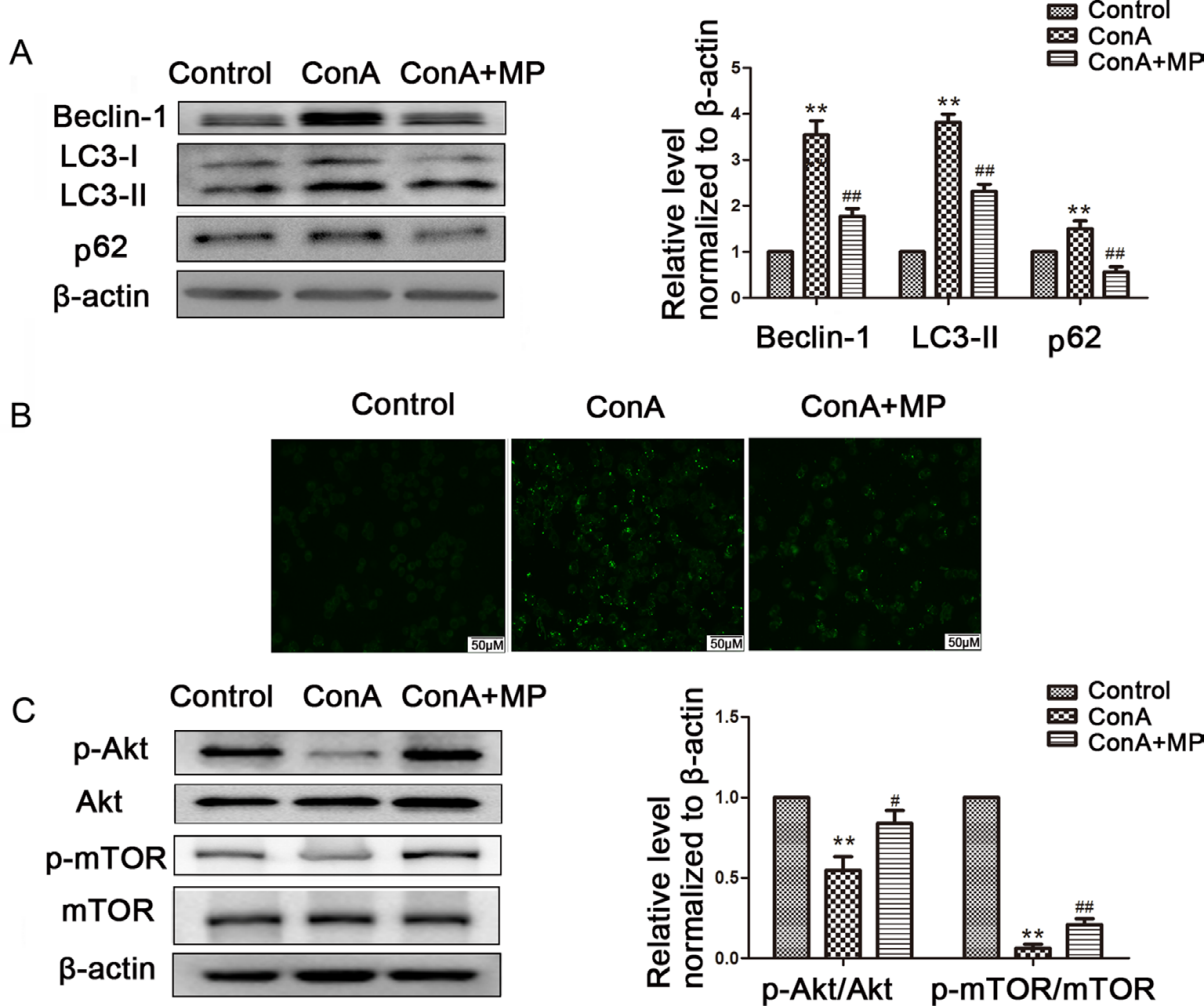
The protective effects of MP treatment on autophagy function in LO2 cells. **(A)** The protein expression of beclin−1, LC3 and p62 was measured by western blot. **(B)** Following each treatment, acidic vacuoles in LO2 cells were stained with MDC and observed under a fluorescence microscope. **(C)** The protein expression of Akt, p-Akt and p-mTOR, mTOR was measured by western blot. β−actin was used as the internal control. The relative protein expression level in the treated group was calculated after setting the control group expression value to 1.00. Data are presented as the mean ± SD. **P < 0.01 vs. control group; ^#^P < 0.05 vs. ConA group; ^##^P < 0.01 vs. ConA group. ConA, concanavalin A; MP, methylprednisolone; MDC, monodansylcadaverine.

The expression of LC3-II, p62, and Beclin-1 was detected by western blot. After pretreatment with 10 μM MP, the expression of autophagy markers in LO2 cells was decreased (**Figure 7A**). Next we used MDC, an acidotropic dye which tends to accumulate in late stage autophagosome like vacuoles to monitor the effect of MP on autophagy (Klionsky et al., 2016). As shown in **Figure 7B**, significantly more MDC-labeled vacuoles accumulated in the cytoplasm of cells in the ConA group than in the control group, while MP pretreatment decreased the accumulation of MDC-labeled vacuoles. To study the regulatory effect of MP treatment on the Akt/mTOR pathway, we examined the levels of phosphorylated Akt1 and mTOR in LO2 cells following MP treatment. MP treatment increased the phosphorylation of AKT1 and mTOR (**Figure 7C**). These *in vitro* data verify that apoptosis and autophagy in hepatocytes are redulated by MP treatment partly through the Akt/mTOR signaling pathway.

## Discussion

AIH is currently viewed as an important component of noninfectious liver disease and it has attracted much attention in recent years, although the etiology and pathogenesis of AIH are still uncertain. Aberrant autoimmunity is thought to induce an interaction between genetic susceptibility and unknown environmental triggers to cause persistent hepatocyte damage. Precision medicine for AIH, including diagnosis, medicinal therapy, and drug monitoring, is a novel therapeutic pattern distinct from evidence-based medicine that aims to develop a personalized treatment strategy (Bossen et al., 2018; Chen et al., 2019; Fan et al., 2019). However, ∼20% of AIH patients exhibit a poor response to standard treatment with glucocorticoids alone or in combination with azathioprine, and the number of effective second-line therapeutic options for AIH is limited (2015; Cropley and Weltman, 2017; Shen et al., 2018). Thus, exploring the mechanisms underlying the effects of known drugs on therapeutic targets is necessary for research and drug development in AIH.

Glucocorticoids are suggested to protect several types of cells, including human fibroblasts and auditory hair cells, from apoptosis (Haake et al., 2009; Nieuwenhuis et al., 2010). Zhao et al. revealed that dexamethasone (DEX) protected LO2 cell hepatocytes from apoptosis induced by a tumor necrosis factor-related ligand that induces apoptosis by upregulating the expression of P-glycoproteins. In addition, several studies have also found an association between the pharmacological mechanism of DEX with up- or downregulated autophagy and apoptosis in other disease models (He et al., 2016; Wang et al., 2018b). In view of these observations, the present study investigated whether MP protects hepatocytes from mitochondria-mediated apoptosis and autophagy dysfunction in EAH mouse livers. ConA injection in mice caused increased cytokines and evident hepatic injuries, which manifested as increased serum levels of transaminases, cytokines, and histopathological changes in the liver. MP treatment alleviated liver damage in mice by increasing the serum levels of ALT and AST. Furthermore, MP treatment inhibited mitochondria-mediated apoptosis and ameliorated autophagy dysfunction in ConA-induced hepatocyte injury at least in part through the Akt/mTOR signaling pathway. The results from experiments in LO2 cells verified these *in vivo* results.

Apoptosis is the predominant mechanism of hepatocyte death in AIH (Czaja, 2014). Interventions that directly modulate apoptosis in AIH have not been studied in the clinic but have been shown to promote malignancy in animals. A number of potential drugs were shown to have protective effects in an AIH model partly through the inhibition of hepatocyte apoptosis (El-Agamy et al., 2018; Feng et al., 2018a; Kim et al., 2018; Yu et al., 2019). Most studies on AIH have found that the antiapoptotic effects of these drugs depend on cytokines secreted by inflammatory cells, including activated T cells and macrophages. In the present study, MP functioned as an antiapoptotic agent that prevented apoptosis in the livers of EAH model mice through a mitochondria-mediated pathway. ConA is a powerful stimulus that triggers an *in vitro* immune response (Taniguchi et al., 1989); Leist *et al*. found that ConA is directly toxic to cultured hepatocytes due to the excessive activation of membrane receptors and subsequent disturbance of the cytoskeleton, which is associated with a strong affinity for the hepatocyte vascular membrane (Leist and Wendel, 1996). Hence, we used direct ConA treatment to mimic the inflammation microenvironment. Our *in vitro* experiments provide unequivocal evidence for the direct protective role of MP in ConA-induced hepatocyte injury.

Autophagy, a lysosomal degradative pathway that is often used to eliminate damaged or unnecessary organelles and intracellular microbial pathogens, can be simulated by various factors, including starvation, growth factor deprivation, and hypoxia. Although autophagy plays a significant role in the pathogenesis of liver disease, its influence on the pathogenesis of EAH remains controversial (Rautou et al., 2010; Puri and Chandra, 2014). Previous studies have found that increased autophagosome formation may be generated by activation of the autophagy process, blockage of autophagy flux caused by the inefficient fusion of autophagosomes and lysosomes, or lysosomal dysfunction (Rothermel and Hill, 2007; Singh et al., 2009). In the present study, the *in vivo* data were obtained using several widely used and complementary approaches to detect autophagy at the tissue level; electron microscopy was to identify autophagic vesicles at the cellular level, and autophagy-related protein expression was evaluated by immunoblotting and immunohistochemistry. An increased number of autophagic vesicles was observed in the hepatocytes of ConA-induced EAH mice, while the expression of p62, a protein degraded by autophagy, was also increased. To provide an overview of the autophagic process, bafilomycin A1, commonly used *in vitro* to verify the patency of autophagy flux, was applied in the subsequent cell experiments. Bafilomycin A1 treatment did not augment the protein expression of LC3-II, indicating that the increased LC3-II expression was not caused by autophagy activation, but blockage of autophagy flux caused by the inefficient fusion of autophagosomes and lysosomes, or lysosomal dysfunction. This finding was in line with several recent studies using immortalized human hepatocytes or liver tissues from patients with liver disease, which implied autophagic protein degradation dysfunction and possible inefficient fusion between autophagosomes and lysosomes (Sir et al., 2008; Rautou et al., 2010; Rautou et al., 2011; Liu et al., 2014). The role of mTOR in the regulation of autophagic flux remains complicated, and the overall effect of mTOR on autophagic flux may largely depend on autophagic flux itself (Zhou et al., 2013). We found significantly increased levels of p-AKT and p-mTOR following MP treatment *in vivo* and *in vitro*. These results indicate that the amelioration of autophagy dysfunction by MP involves recovery of the Akt/mTOR signaling pathway; that is, activation of mTOR is correlated with the improvement of autophagic flux. Our data are in line with previous studies, which revealed that the activation of mTOR can accelerate the elimination of autophagic vacuoles by enhancing the function of the intracellular autophagy pathway in cells (Bains et al., 2009; Hu et al., 2015).

It should be noted that the present study had several limitations. First, the mechanisms involved in immune-induced liver injury are complex, and ConA-induced hepatocyte injury could partially mimic *in vivo* mechanisms rather than human AIH (Hardtke-Wolenski and Jaeckel, 2010; Ye et al., 2018; Christen, 2019). A few previous studies used ConA stimulation in hepatocytes or cell lines to explore the detailed mechanisms of immune-mediated liver injury (Chen et al., 2014; Li et al., 2016a; Ko et al., 2018; Yang et al., 2019), and ConA stimulation has also been applied to study monocytes and macrophages in immune reactions (Chanput et al., 2010; Zhuang et al., 2016; Nakamoto et al., 2017; Shi et al., 2019). Thus, this study aimed to evaluate the direct molecular mechanism of MP treatment on ConA-induced hepatocyte injury, and we believe that the *in vitro* data are relevant to the *in vivo* data. Further research is required to elucidate the exact mechanism of ConA−induced liver injury. Second, we did not explore more detailed mechanism of impaired autophagy flux during the process. Third, the present study was performed using mice and cellular experiments, and additional studies on human AIH are needed to verify the protective effects in the future.

In conclusion, both *in vivo* and *in vitro* experimental data suggest that MP treatment ameliorates mitochondria-mediated apoptosis and autophagy dysfunction, which is associated with the Akt/mTOR pathway. The current study suggests the clinical and rational use of MP to treat AIH. However, the detailed molecular mechanisms of the effect of MP on hepatocytes in AIH are not completely clear. These results provide insights into the mechanisms underlying the effect of MP on hepatocytes.

## Data Availability Statement

The raw data supporting the conclusions of this manuscript will be made available by the authors, without undue reservation, to any qualified researcher.

## Ethics Statement

The animal study was reviewed and approved by Institutional Animal Care and Treatment Committee of Sichuan University.

## Author Contributions

XF and RM wrote the manuscript. LY and XL designed the research. TY, XF, RM, and HW performed the research and analyzed the data. TY, MS, and TW participated in the research design. XF performed the data analysis.

## Funding

This study was funded by a grant from the National Natural Science Foundation of China (no. 81770568 to Li Yang) and the 1.3.5 project for disciplines of excellence,West China Hospital, Sichuan University (ZYJC18008).

## Conflict of Interest

The authors declare that the research was conducted in the absence of any commercial or financial relationships that could be construed as a potential conflict of interest.
