# Supplementary material for: Methylprednisolone Decreases Mitochondria-Mediated Apoptosis and Autophagy Dysfunction in Hepatocytes of Experimental Autoimmune Hepatitis Model via the Akt/mTOR Signaling
Source: Front Pharmacol. 2019 Oct 18;10:1189. doi: 10.3389/fphar.2019.01189 (PMC6813226; doi:10.3389/fphar.2019.01189)
Supplement: Supplementary file 1 [file DataSheet_1.docx]

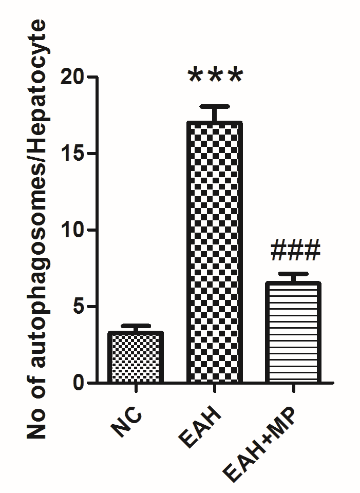


Supplementary figure 1

Supplementary figure 1. The amount of autophagosomes in hepatocytes of the hepatic tissues in Figure 3C. ***P<0.001 vs. NC group; ###P<0.001 vs. EAH group.


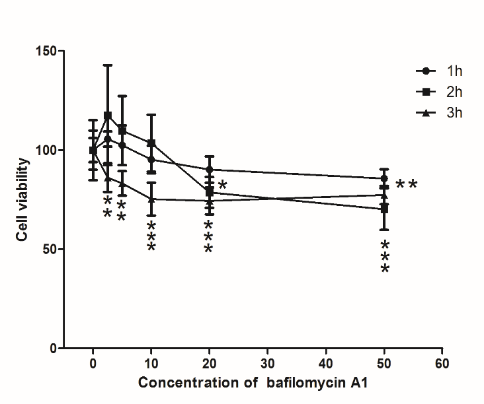


Supplementary figure 2

Supplementary figure 2. The effects of bafilomycin A1 on hepatocytes cell viability. Hepatocytes were treated with increasing doses of bafilomycin A1 for 1h, 2h and 3 h, respectively. Each point represents the mean ± SD for three independent experiments.*p < 0.05, **p < 0.01, ***p < 0.001 vs. vehicle control.
